# A Cross-Sectional Survey of National Chinese College Students’ Mental Status during COVID-19 Pandemic: Using a Compiled Stress Response Questionnaire

**DOI:** 10.3390/ijerph191912079

**Published:** 2022-09-24

**Authors:** Ying Guo, Hongyu Zhang, Yufei Xie, Xin Tian, Na Luo, Yan Zhang

**Affiliations:** 1 School of Psychology, Sichuan Normal University, Chengdu 610066, China; 2 The College of Liberal Arts and Sciences, Arizona State University, 1151 S. Forest Ave., Tempe, AZ 85281, USA; 3 Department of Orthodontics, Shanghai Xuhui District Dental Disease Prevention and Control Institute, Shanghai 200001, China; 4 School of Educational Science, Huazhong University of Science and Technology, Luoyu Road No. 1037, Hongshan, Wuhan 430074, China

**Keywords:** college students, mental health, psychological stress, COVID-19, pandemic situation

## Abstract

As the coronavirus disease (COVID-19) spread, local governments in China adopted severe lockdown measures to control the pandemic. People were restricted from traveling for leisure, business, education, and medical treatment. Changes were observed in people’s psychological states as a result of COVID-19. This study aimed to construct a Stress Response Questionnaire based on the stress response theory for use with college students, to access their authentic mental health conditions and provide psychological intervention suggestions and countermeasures for the emergency plan formulated by the education department. The questionnaire was used to collect responses from a sample of 16,353 college students from 34 provinces in China. The results showed that anxiety levels varied by sex, current location, and pandemic region, each of which predicted different levels of emotional disturbance. Thus, we highly recommend that the Chinese college administration implement appropriate intervention programs and procedures to help college students maintain their mental health.

## 1. Introduction

The COVID-19 pandemic has caused a dramatic loss of human life, posing unprecedented challenges to public health, food systems, and the workplace. More than 500 million laboratory-confirmed cases were documented as of 26 July 2022 [1]. Amid the highly contagious characteristic, unknown pathogenesis, and unclear modes of transmission of COVID-19 in 2020, prevention and control studies were hardly conducted. Meanwhile, the Chinese government adopted severe control measures as the disease spread, which caused panic among the public [2]. The pandemic has had a negative impact both on people’s physical and mental health. In one study, an estimated one in five participants suffered from depressive symptoms and sleep disorders [3]. In addition, acute stress disorder (ASD) and post-traumatic stress disorder (PTSD) may be predicted by media exposure during the pandemic [4], as the Internet has become the primary source of information related to COVID-19 [5]. This created an enormous challenge for the existing mental health-care system in China.

In the wake of COVID-19, people have experienced significant disruptions to their work and daily lives. Many college students have even experienced stress responses. Additionally, the concept of stress was proposed by Selye, who believed that stress was the sum of an individual’s non-specific responses to external or internal stimuli, accompanied by psychological experiences of tension [6]. Stress measurement tools commonly used in existing studies are the PTSD Check List—Civilian Version (PCL-C), the Stress Response Questionnaire (SRQ), and the Perceived Stress Scale (PSS-10). PCL-C identifies four dimensions of post-traumatic stress disorder: hyperarousal, avoidance, reexperiencing, and numbing/dysphoria [7]. SRQ utilizes psychological stress theory to develop a scale that determines how much psychological stress is present in an individual along with their physical and mental symptoms. Three dimensions are included in the scale: emotional, physical, and behavioral [8]. Further, PSS-10 is the most widely used tool for evaluating stress perception, which incorporates both negative and positive feelings [9]. As can be seen, existing tools for measuring stress response are often based on symptom screening indicators, primarily concerned with physical and psychological health, instead of incorporating the concept of a well-functioning social life into mental health.

The stress response, according to the stress response theory, is a physiology–psychology–behavioral response caused by a combination of stressors and various mediators [10]. The event that an individual perceives as a threat to himself or herself is referred to as a stressor. The physiology–psychology–behavioral response is primarily concerned with changes in psychological, physiological, and behavioral indicators [10]. Such changes include anxiety, depressed mood, and other psychological indicators; hypertension, insomnia, headaches, and other physiological indicators; and avoidance, aggression, confrontation, and other behavioral indicators, the most obvious of which are emotional changes. Modest stress enhances the body’s ability to adapt [11]. However, chronic and uncontrollable stress can lead to a state of extreme discomfort physically and mentally in response to life-threatening events, such as war, violence, etc. The results of a meta-analysis showed that under the COVID-19 pandemic, Chinese college students showed stress responses such as lack of energy, lack of pleasure, sleep disorder, staying up late, and irregular bedtimes [12]. ASD and PTSD, which are exemplified by nightmares, flashbacks, or intrusive recollections of traumatic events [13], may have developed during the pandemic [14]. Under sustained stress, most people also show impaired social functioning, with abnormalities in their interpersonal interactions and social lives [15]. Coping style is also an essential factor contributing to stress response since it represents an individual’s cognitive and behavioral efforts to cope with distress. Coping can be divided into cognitive and behavioral coping, and the social functioning of a person may be adversely affected by inappropriate coping styles [16]. In conclusion, based on the stress response theory, individuals will adopt different coping styles in the face of sudden and dangerous events and are prone to stress reactions, accompanied by changes in social functioning. 

The uncertain factors brought by COVID-19, such as increased employment difficulty, limited learning opportunities, and restricted life, have put college students under a variety of pressures for a long time, which has also had a huge impact on their physical and mental health [17,18]. Measures of “home isolation” have also led college students to show excessive attention to social media platforms such as Weibo, WeChat, and TikTok, a behavior that further exacerbates public mental health problems [19,20]. Excessive media exposure also led to a significant increase in the incidence of online violence during the pandemic, which, in turn, affected the normal social interactions of college students [21]. In addition, the unstable psychological state of college students makes the impact of the pandemic more prominent for them [22,23]. National attention toward the psychological impact of COVID-19 on Chinese college students is necessary because college students are significant components of Chinese society, and their mental states affect the shaping of social development [24].

Accordingly, college students were selected as the research sample in this study. Based on the stress response theory, we took three important aspects (stress response, coping style, and social function) into consideration, and compiled the Stress Questionnaire for college students in the context of the COVID-19 pandemic. Additionally, using the questionnaire, we investigated the psychological health of college students during the pandemic. The aim is to provide evidence for a thorough understanding of the mental health status of college students during the pandemic. It also provides a reference for Chinese health and education departments to make mental health promotion plans and carry out psychological interventions.

### Research Overview

This study is divided into two sub-studies. For Study 1, we created a Stress Response Questionnaire for college students. Its reliability and validity were confirmed. Study 2 examined the mental health of college students during the COVID-19 pandemic in 34 Chinese provinces. The study was also designed in accordance with the principles of the Declaration of Helsinki. It was approved by the ethical authority of the School of Educational Sciences, Huazhong University of Science and Technology. Anonymous codes were assigned to the self-report questionnaires to manage confidentiality. From 29 January to 2 February, information about college students was gathered through an online questionnaire (www.wjx.cn) [25,26]. The questionnaires were distributed across social media platforms in China (e.g., WeChat, QQ, and Weibo). We asked all participants for informed consent before they started the questionnaire, and we asked for their socio-demographic information. They then filled out questionnaires about their psychological status.

## 2. Study 1: Compilation of Stress Response Questionnaire for College Students

### 2.1. Participants and Compilation for Questionnaire

#### 2.1.1. Participants

Sample 1: We collected questionnaires from 2046 college students. The factors were determined using exploratory factor analysis (EFA). Sample 2: We compiled a Stress Response Questionnaire that was tested by 2030 college students, and the final version of the questionnaire was proven to have good reliability and validity via confirmatory factor analysis (CFA). A total of 247 ineffective questionnaires were removed, possibly because respondents completed the questionnaire within 5 min or answered the same way for eight or more consecutive questions [27].

#### 2.1.2. Compilation of Stress Response Questionnaire

This study theoretically constructed a Stress Response Questionnaire for college students based on the stress response theory and actual impacts on college students caused by COVID-19. In addition, the questionnaire compilation examined three important aspects: stress response, coping style, and social functioning. During the pandemic, the researchers interviewed college students and administered open-ended questionnaires to assess their mental health. Finally, 32 items were created after clarifying or removing items with unclear expressions or ambiguous contents. In addition, this questionnaire employed a 5-point scale, with 1 representing the least severe and 4 or 5 representing the most severe. The socio-demographic information section included age, sex, educational status, relationship status, and health status (whether or not participants were infected with COVID-19). 

### 2.2. Statistical Methods

SPSS 22.0 and AMOS 21.0 were used to conduct exploratory factor analysis and reliability and validity tests on the data.

### 2.3. Results

#### 2.3.1. Exploratory Factor Analysis (EFA)

The Kaiser–Meyer–Olkin (KMO) value test and Bartlett’s test of sphericity were applied to the prediction data of the questionnaires to confirm the suitability of the data for EFA. In the tests performed, both KMO, which had a value of 0.925 (*χ*^2^ = 24,502.18, df = 496; *p* < 0.01), and Bartlett’s test of sphericity were significant, indicating that the data of the questionnaire met the criteria for EFA. Furthermore, combining principal component analysis with the gravel plot test yielded seven factors with eigenvalues greater than 1, and the cumulative variance explanation rate was 55.92%. Considering that the factor loadings ranged from 0.45 to 0.87, the structural validity of this scale was acceptable [28,29]. Table 1 presents the results of factor loadings.

Finally, EFA indicated that the questions included three general dimensions, which are described in detail below.

First, the stress responses, which are composed of emotional, somatic, and behavioral responses, included 12 items (Q1, Q2, Q3, Q4, Q5, T1, T2, X2, R5, J4 X3, and Y5). The meaning of each item is as follows: Q1 (Have I felt more nervous and anxious than usual in the past week?), Q2 (I have been scared for no reason for the past week), Q3 (I have been easily upset or panicked in the past week), Q4 (I have been feeling sullen and depressed for the past week), Q5 (I have felt uneasy for the past week and can’t calm down), T1 (I have felt like I was losing weight for the past week), T2 (I have been tired for almost a week for no reason), X2 (I am still interested in the things that I used to be in the past week), R5 (I think strangers are carrying the COVID-19 virus), J4 (I dare not speak to strangers), X3 (I forced myself to wash or clean multiple times, thinking it was full of germs and viruses), and Y5 (I desperately want to go to the hospital for a checkup). 

Second, social functioning, which consists of life disturbance, interpersonal disturbance, and emotional disturbance, included seven items (X1, Y4, J1, J2, Y2, Y1, and Y3). The meaning of each item is as follows: X1 (I have a hard time concentrating on things other than viruses), Y4 (I am so worried about being infected that it affects my normal work and rest), J1 (The emergence of COVID-19 has an impact on my interpersonal relationship), J2 (I feel distant from or disconnected from crowds), Y2 (It was difficult to calm down last week), Y1 (I feel like I’m getting irritable or short-tempered), and Y3 (I am very troubled by this pandemic).

Finally, coping style, which consists of risk coping, protective coping, information coping, stressful coping, and looking for social support, included 12 items (R1, R2, R3, F2, F1, R4, G1, L4, L1, L5, L2, and L3). The meaning of each item is as follows: R1 (I think the pandemic is very serious now), R2 (I think I am in danger of COVID-19.), R3 (I think it is very likely that I have COVID-19), F2 (I think village-to-village (community-to-community) isolation is necessary), F1 (I think that as long as we take protective measures (wearing a mask when going out, washing hands frequently, and disinfecting regularly), we will not be infected), R4 (I think COVID-19 can be cured), G1 (I have been paying close attention to the school’s official information in the past week), L4 (I have been looking for psychological intervention resources for the past week to make self-adjustment), L1 (The sudden pandemic has made me very stressed), L5 (I feel like I’m becoming suspicious), L2 (I am actively looking for ways to relieve stress), and L3 (I am happy to tell my troubles to those close to me).

#### 2.3.2. Reliability Test

Internal consistency reliability (Cronbach’s alpha coefficient) and split-half reliability (Spearman–Brown) were employed in this study to test reliability on the basis of 2030 valid data points. The study results indicated that the overall internal consistency coefficient of the questionnaire was 0.872 and its overall split-half reliability was 0.736. These values meant that the questionnaire prepared in this study had positive reliability and could be used as a reliable research measurement tool.

#### 2.3.3. Validity Test

**Figure 1:**
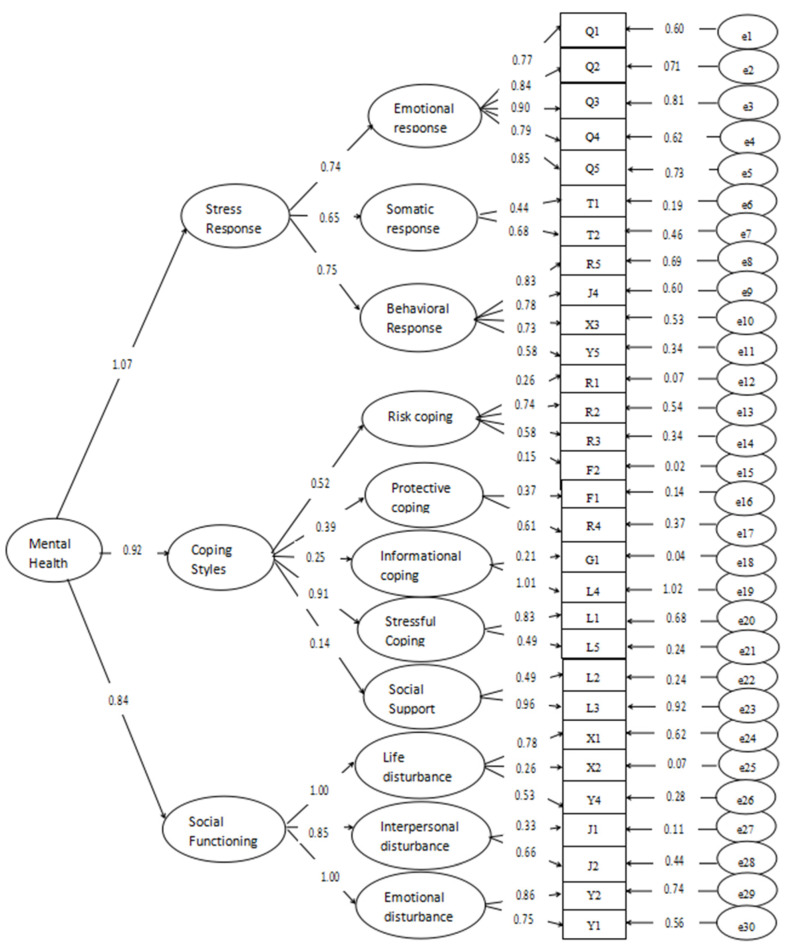
Confirmatory factor analysis of model of college students’ mental health during the pandemic.

CFA and correlation analysis were used to evaluate the construct validity of the questionnaire. According to the results of the EFA, the factors were named after deleting an item that could not be classified (J3). To adjust the items and factors, we used the stress response theory and the theoretical conception of the questionnaire structure, which allowed us to summarize the 31 items into 11 named factors. Using 2030 valid questionnaires, AMOS was used to perform CFA on the valid questionnaires. The Y3 item was removed during the process of constructing and debugging the model. As shown in Figure 1, the final measurement model consisted of 30 items. In this study, the model fitting index results showed that χ^2^/df = 5.916, CFI = 0.910, GFI = 0.925, NFI = 0.894, and RMSEA = 0.049, and the latent variable model constructed by the 30 items in the EFA had well-fitted data structure.

An analysis of the correlation between dimensions was used to estimate the construct validity of the questionnaire. In Table 2, the correlation coefficients between the questionnaire dimensions were all less than 0.69, indicating that they were all statistically significant. The study results indicated good discrimination between the various dimensions of the questionnaire. Furthermore, the variables appeared to have some degree of independence and a relationship between them. Accordingly, the CFA revealed that the self-made questionnaire had an ideal structure validity based on the moderately significant correlation between the dimensions.

In summary, the Stress Response Questionnaire for college students developed in this study consisted of three dimensions (stress response, coping styles, and social functioning), with a total of 30 items. Among them, stress response consisted of emotional, somatic and behavioral responses, and included 11 items. Coping styles consisted of risk coping, protection coping, informational coping, stressful coping, and social support, and included 12 items. Social functioning consisted of life disturbance, interpersonal disturbance, and emotional disturbance, and included seven items. The indicators of the scale satisfy the psychometric requirements, with good reliability and validity, and can be used as a measurement tool for subsequent related research. 

## 3. Study 2: Mental Health Survey of College Students during COVID-19

### 3.1. Participants and Measurement Questionnaire

The final version of the Stress Response Questionnaire for college students during the pandemic was used. As a result, 16,353 effective questionnaires were collected from college students from 34 provinces and autonomous regions of China (Hong Kong and Macao) between 29 January and 2 February 2020. Overall, 7850 females and 8503 males participated in the survey. The participants consisted of 13,084 undergraduates, 219 graduate students, 42 PhD students, and 3008 vocational students. Their average age varied from 19.79 ± 1.96 years. Ultimately, the investigations resulted in an effective response rate of 98.5%.

### 3.2. Statistical Methods

SPSS 22.0 was used to perform descriptive statistics and an independent samples *t*-test.

### 3.3. Results

#### 3.3.1. Results of Descriptive Statistics

The results of the descriptive statistics showed that 48.4% of students were located in Wuhan (urban and rural areas), while 51.6% were located elsewhere in China. In addition, 40.89% of the questionnaires returned were from students living in cities near Wuhan, thus suggesting that nearly half of the students lived in Hubei province, the epicenter of the pandemic. Furthermore, 54.2% of all students came from townships (rural areas) and 45.8% from cities (urban areas); additionally, 48% of the questionnaire respondents were females, while males comprised 52%. A total of 78.8% of all college students were single, 20.8% were in a relationship, and only 0.4% were married.

Based on descriptive statistics, it was determined that the mean scores for emotional responses, physical responses, behavioral responses, life distress, interpersonal distress, and emotional distress were all lower than the theoretical mean (M = 2.41), thus indicating that college students showed weaker stress responses to the pandemic. The students had strong protective factors against the pandemic and were able to adopt protective measures effectively, as the average scores of risk coping, protective coping, stress coping, and seeking social support all exceeded the theoretical mean. The information coping rating was below the hypothetical average, which may be related to college students’ excessive attention to pandemic-related information.

#### 3.3.2. Differences in Demographic Variables

In this study, independent samples *t*-tests were conducted to test the significance of differences in the three demographic variables of sex, current location, and region for each dimension of psychological stress during the pandemic among college students (see Table 3). The results indicated that sex, current location, and current region (urban and rural) significantly influenced the dimensions of the questionnaire. That is, the three social backgrounds had different degrees of impact on mental health.

##### Sex Differences in Stress Responses

The results in Table 3 of the independent samples *t*-tests on the collected data indicate that females tended to show more emotional responses (*t* = −11.73, *p* < 0.001) and behavioral response (*t* = −5.00, *p* < 0.001) than males, and preferred to adopt risk coping (*t* = −24.15, *p* < 0.001), protective coping (*t* = −3.78, *p* < 0.001) and stress coping (*t* = −11.68, *p* < 0.001) when encountering stress, whereas males preferred informational coping (*t* = 9.89, *p* < 0.001). However, females tended to seek social support, contrary to males (*t* = −19.61, *p* < 0.001), who seemed to meet more interpersonal disturbances than females (*t* = 5.93, *p* < 0.001) according to the data results. There were no significant sex differences in somatic response, life disturbances, and emotional disturbances.

##### Current Location Differences in Stress Responses

The survey revealed that students who were inside Hubei province showed more behavioral responses (*t* = 7.82, *p* < 0.001) and somatic responses (*t* = 2.14, *p* < 0.05) rather than emotional responses (*t* = −2.35, *p* < 0.05) (Table 3). We also found that students outside of Hubei province were more positive in adopting protective coping (*t* = −2.63, *p* < 0.01), informational coping (*t* = −2.63, *p* < 0.01), stress coping (*t* = −2.45, *p* < 0.05), and in seeking social support (*t* = −3.64, *p* < 0.001). However, students who were inside Hubei province faced more life disturbances (*t* = 8.65, *p* < 0.001) and emotional disturbances (*t* = 6.68, *p* < 0.001) compared to those outside.

##### Current Regional Differences in Stress Responses

Regarding the dimension of the current region (rural or urban areas), students who came from rural areas reported fewer emotional (*t* = −5.10, *p* < 0.001) and somatic responses (*t* = −7.60, *p* < 0.001), whereas they chose to take behavioral responses (*t* = 2.95, *p* < 0.01) in responding to the pandemic. Students in rural areas reported more protective coping (*t* = 5.29, *p* < 0.001) and informational coping (*t* = 3.96, *p* < 0.001) but less seeking of social support (*t* = −7.33, *p* < 0.001). There is also a significant difference in social functioning, since students in urban areas reported fewer life disturbances (*t* = 9.09, *p* < 0.001) and interpersonal disturbances (*t* = 2.68, *p* < 0.01).

## 4. Discussion

### 4.1. Compilation of Stress Response Questionnaire for College Students

The present study first made predictions based on the initial questionnaire, and then, performed an EFA of the valid data. After one item that could not be classified was eliminated, the extracted factors could explain 55.92% of the variance. The prepared questionnaire was also investigated to ensure its reliability. In the reliability test, split-half reliability was used as the reliability index, and the results showed that the questionnaire had good reliability. The validity of the questionnaire was tested via correlation analysis and CFA, which indicated moderately significant correlations among the dimensions, and the results of the CFA analysis also confirmed the ideal structural validity of the Stress Response Questionnaire for college students.

By combining top-down theoretical analysis with bottom-up empirical investigation, this study found that the Stress Response Questionnaire administered to college students during the COVID-19 pandemic incorporated emotional, physical, and behavioral stress; life distress; interpersonal disturbances, etc. Individuals may experience physiological, emotional, and behavioral changes as a result of stress. Therefore, three dimensions were considered: (a) emotional response, (b) physical response, and (c) behavioral response. The factors mediating the relationship between stress and stimuli were also considered. Coping style may be regarded as an example of a mediating variable, which is essential in understanding the consequences of life distress [30]. Risk coping, information coping, and stress coping are all cognitive responses, whereas protective responses and seeking social support are behavioral responses. Individuals are likely to express acute stress responses in both challenged and threatened states [31], accompanied by cognitive and behavioral coping strategies. This research constructs a Stress Response Questionnaire for college students during the pandemic based on the theory of stress response and the occurrence mechanism of psychological processes, which has certain innovative value and highly practical implications.

### 4.2. Differences in Demographic Variables

#### 4.2.1. Sex Differences in Responding to the Pandemic

In terms of sex, females had a more robust stress reaction to the pandemic than males [32,33] and usually had a stronger sense of danger. These findings are consistent with previous studies in which women have significantly higher rates of ASD and PTSD than men in emergency situations, and that women are also more likely to experience more psychological stress from depression, anxiety, stress, and other stress responses [14,29,34]. This reaction may be due to sex differences in brain activity. A study found that chronic social isolation stress changes key brain regions involved in emotion control, such as the prefrontal cortex (PFC), basal lateral amygdala (BLA), and ventral tegmental area (VTA) [35]. On this basis, the researchers also found that chronic stress exposure usually resulted in the triggering of depression-like emotional responses in female rodents, but not in males [36]. In addition, it is found that after adolescence, females begin to have higher levels of emotional empathy than males and are more susceptible to the effects of negative emotional events [37]. There were also significant sex differences in emotional processing, mainly in terms of females having an emotional recognition advantage, a better emotional memory ability, and greater susceptibility to negative emotions [38].

Meanwhile, males were more likely to respond rationally to the pandemic, and they reached the goal of protecting themselves by paying close attention to the information. Tracing the evolutionary roots of sex differences, male resources and status are closely tied to mate choice in order to better survive and reproduce [39]. Males who can provide resources and protection are more likely to be selected, and thus, the way males behave is closely related to their social roles and has some evolutionary significance. However, males were more inclined to show discomfort in interpersonal disturbance during the pandemic, which is consistent with previous findings [36]. In terms of neurophysiological systems, hyperactivity of the basolateral amygdala in male mice under chronic social isolation stress led to increased aggression [40]. In conclusion, males are more likely to show close attention to information and interpersonal disturbance during the pandemic. 

#### 4.2.2. Current Location Differences in Responding to the Pandemic

In the course of the massive pandemic, regional differences existed in the psychological health of the general public [5]. In Hubei province, severe stress responses were mainly characterized by somatic and behavioral manifestations, following the current location factor. People were suddenly and severely restricted from traveling, shopping, and engaging in certain forms of entertainment. Moreover, social distancing was strictly enforced (to prevent the spread of the virus [41]). These policies had profound effects on people’s daily lives. However, students outside of Hubei province also showed higher emotional responses and adhered to coping strategies more than students inside of Hubei did, which may be due to the typhoon eye effect, in which perceptions of risk events at the epicenter are lower than the perceived risk in surrounding areas [26]. It can also be explained by the ripple effect. The ripple effect refers to the effect of risk events that are amplified through media publicity or other unofficial channels and affect the lives of people in peripheral areas. Given the lack of direct experience and information, people in these regions only accept amplified information and develop a higher perception of risk [42].

#### 4.2.3. Current Regional Differences in Responding to the Pandemic

Higher emotional and physical stress responses were observed in urban students than in rural students. This difference may be due to the urban population density. The main routes of transmission of the virus are airborne droplet transmission and close contact transmission. Since cities have a larger population base and a higher likelihood of infection with the virus, the urban residents showed a higher emotional and somatic response. However, in the countryside, transportation, networks, and other facilities were not as reliable as in cities, resulting in a reduced flow of information, increased behavioral reactions, and a more significant impact on interpersonal relations for students. In addition, due to the lack of internet access in rural areas, people’s interactions relied more on face-to-face interactions. The pandemic made it necessary to maintain a certain distance from others in human interactions, which, in turn, exacerbated the life distress and interpersonal distress of rural residents and had a more significant impact on students’ interpersonal relationships.

## 5. Conclusions

By investigating the mental health status of Chinese college students after the COVID-19 outbreak, it was found that female students had a stronger stress response than male students. The emotional response of college students in areas outside the pandemic center was slightly higher than that of students in the pandemic center, and they used social media to track the development of the pandemic more. In addition, students in rural areas had fewer emotional and somatic responses than those in urban areas because the countryside is far away from densely populated areas, where the pandemic was not easily developed. The government and university workers need to attach great importance to the mental health development of students and make timely interventions. It should be noted that although the data in this study came from the early stage of the pandemic, it is still relevant to comprehensively grasp the mental health status of college students and carry out targeted psychological counseling and educational guidance work at a time when the pandemic is normalized.

## 6. Suggestions

### 6.1. Adopting Effective Psychological Interventions

Effective interventions can change people’s mindsets about stress [43]. Research has found that exposure-based therapy can effectively reduce subsequent PTSD symptoms to a greater extent in patients diagnosed with ASD. PTSD symptoms can also be reduced by prolonged exposure [44]. Using it early can prevent high-risk ASD people from developing PTSD [45]. Finally, cognitive behavior therapy and mindfulness-based therapy can also combat anxiety and prevent depression [46]. Therefore, effective psychological interventions should be chosen by psychological professionals. Additionally, to acquire professional and systematic treatment for college students in need, schoolteachers should refer them to a mental health center or provide access to teletherapy [47].

### 6.2. Implementing Effective Education Management

Educators can take several approaches to help students manage their stress. First, licensed mental health teachers can conduct cognitive enhancement practices to make students recognize why stress management is significant to their long-term health conditions (mentally and physically) and their later generations. Second, courses related to mental health and effective thinking are also necessary. As a result of widespread misleading information, effective thinking courses would help students identify false and true information. When citizens cannot think critically and independently, they are easy prey for dogmatists, film artists, and those who offer simple solutions to complex problems [48]. Moreover, universal mental health promotion programs should be developed by related departments in schools since those long-term mental health interventions may be more productive than brief, class-based mental illness-prevention programs [49].

### 6.3. Development of Social Support Systems

In general, a social support system is the most significant resource for emotional recovery from a catastrophe [50], and stress-related anxiety symptoms may be alleviated by social support [51]. A strong social support system can contribute to the mental well-being of an individual. According to one study, social support is helpful for the treatment of PTSD [52]. Another study shows that psychological distress was significantly reduced by increased social support [53,54]. Importantly, the perception of support appears to be more influential than the actual support [55]. Consequently, the social support system could help college students reduce their stress and build mental resilience against COVID-19.

First, the mental and physical health of students should be thoroughly assessed in schools by supporting the students, staff, and faculty. Second, the school administration should engage psychological counselors to gather basic information, conduct hierarchical screening, provide accurate assistance, and intervene promptly [29]. Third, a program of hotline services could be set up by psychiatric institutions, colleges’ counseling centers, mental health associations, and academic societies [2]. This program would provide external support channels for students seeking outside assistance from professionals. Finally, building harmonious relationships with families and friends should be encouraged by educators, which may be a critical protective factor for the mental health of an individual during the pandemic. As a result, college students may benefit greatly from the emotional support of their families [56].

## 7. Limitations

This study also has some limitations. First, the cross-sectional design of the study showed the correlation between the pandemic and the psychological state of college students. However, researchers should examine the extent of the pandemic’s impact on the mental health of college students in future longitudinal or interventional studies. Second, all the questionnaires we received were anonymous, and one limitation of the questionnaire is its subjectivity. Respondents may still be affected by social desirability, that is, showing behaviors that society hopes for, expects, and accepts. China is a country that particularly emphasizes collectivism [57], nationalism [58], and patriotism [59]. Finally, we investigated the subjective psychological states of college students but did not conduct observations from a third-party perspective. Human mental states are always closely related to behaviors, just as attitudes are often highly correlated with behaviors [60]. More research is needed to track the extent of the pandemic’s psychological impact.

## Figures and Tables

**Table 1:** Factor loadings for each entry (*n* = 2046).

Stress Response			Coping Styles			Social Functioning		
Factor	Items	Loadings	Factor	Items	Loadings	Factor	Items	Loadings
Emotional	Q3	0.842	Risk	R2	0.639	Life disturbance	X1	0.458
	Q5	0.831		R1	0.569		X2	0.546
	Q4	0.798		F2	0.554		Y4	0.402
	Q2	0.759		R3	0.504	Interpersonal disturbance	J1	0.424
	Q1	0.744	Protective	F1	0.713		J2	0.308
Somatic	T2	0.630		R4	0.706		J3	−0.116
	T1	0.459	information	G1	0.740	Emotional disturbance	Y1	0.522
Behavioral	R5	0.812		L4	0.701		Y2	0.469
	J4	0.799	Stress	L1	0.432		Y3	0.406
	X3	0.745		L5	0.371			
	Y5	0.624	Social support	L2	0.835			
				L3	0.785			

**Table 2:** Correlation matrix between dimensions of the questionnaire (*n* = 2030).

	Emotion Response (ER)	Somatic Response (SR)	Behavior Response (BR)	Risk Coping (RC)	Protective Coping (PC)	Information Coping (IC)	Stress Coping (SC)	Seeking Social Support (SSS)	Life Disturbance (LD)	Interpersonal Disturbance (ID)	Emotional Disturbance (ED)
ER	1										
SR	0.507 **	1									
BR	0.458 **	0.254 **	1								
HR	0.324 **	0.093 **	0.250 **	1							
PC	0.168 **	0.071 **	0.151 **	0.174 **	1						
IC	0.196 **	0.114 **	0.101 **	0.040	−0.042	1					
SC	0.527 **	0.311 **	0.469 **	0.280 **	0.146 **	0.100 **	1				
SSS	0.051 *	−0.027	0.097 **	0.061 **	−0.080 **	0.152 **	0.135 **	1			
LD	0.419 **	0.275 **	0.569 **	0.141 **	0.122 **	0.079 **	0.430 **	0.052 *	1		
ID	0.381 **	0.279 **	0.412 **	0.146 **	0.122 **	0.102 **	0.373 **	0.004	0.410 **	1	
ED	0.552 **	0.385 **	0.587 **	0.171 **	0.155 **	0.118 **	0.524 **	0.041	0.629 **	0.488 **	1

Note. * *p* < 0.05, ** *p* < 0.01; the same below.

**Table 3:** Independent samples *t*-tests on demographic variables of psychological stress in college students (*n* = 16,353).

Dimensions	M ± SD	*t*		
		Sex (Male/Female)	Current Location (Inside Hubei Province/Outside Hubei Province)	Current Region (Rural/Urban Area)
Emotional response	1.49 ± 0.57	−11.73 ***	−2.35 *	−5.10 ***
Somatic response	1.30 ± 0.46	−0.85	2.14 *	−7.60 ***
Behavioral response	2.22 ± 0.82	−5.00 ***	7.82 ***	2.95 **
Risk coping	3.51 ± 0.55	−24.15 ***	1.1	0.20
Protective coping	3.66 ± 0.78	−3.78 ***	−2.63 **	5.29 ***
Informational coping	1.73 ± 0.50	9.89 ***	−2.63 **	3.96 ***
Stress coping	2.71 ± 0.92	−11.68 ***	−2.45 *	−0.86
Seeking social support	3.35 ± 0.91	−19.61 ***	−3.64 ***	−7.33 ***
Life disturbance	2.37 ± 0.74	1.83	8.65 ***	9.09 ***
Interpersonal disturbance	2.13 ± 0.81	5.93 ***	1.49	2.68 **
Emotional disturbance	2.08 ± 0.86	−0.96	6.68 ***	1.68

Note. * *p* < 0.05, ** *p* < 0.01, *** *p* < 0.001.

## Data Availability

Data will be stored in a publicly accessible repository and will be available upon publication from the osf.io database (osf.io/saj3p).
